# Draft Genome Sequence of the Anaerobic Arsenite-Oxidizing *Halomonas* sp. Strain ANAO-440, Isolated from an Alkaline Saline Lake in Khovsgol, Mongolia

**DOI:** 10.1128/MRA.00899-21

**Published:** 2021-10-21

**Authors:** Natsuko Hamamura, Narantuya Damdinsuren, Nobuyoshi Nakajima, Shigeki Yamamura

**Affiliations:** a Department of Biology, Faculty of Science, Kyushu University, Fukuoka, Japan; b Department of Biology, School of Arts and Sciences, National University of Mongolia, Ulaanbaatar, Mongolia; c Biodiversity Division, National Institute for Environmental Studies, Tsukuba, Ibaraki, Japan; d Regional Environment Conservation Division, National Institute for Environmental Studies, Tsukuba, Ibaraki, Japan; University of Southern California

## Abstract

The draft genome sequence of *Halomonas* sp. strain ANAO-440 contains 3,866 predicted protein-coding sequences. This strain is capable of anaerobic arsenite oxidation and encodes an *arxA*-type arsenite oxidase within the *arxB2AB1CD* gene island. This genome sequence provides valuable information regarding the physiological diversity of Arx-dependent arsenite-oxidizing microorganisms.

## ANNOUNCEMENT

*Halomonas* sp. strain ANAO-440 is a heterotrophic arsenite-oxidizing halophile isolated from an alkaline saline lake located in the northern region of Khovsgol, Mongolia (1). Strain ANAO-440 was able to oxidize arsenite anaerobically coupled to nitrate reduction and was shown to contain an *arxA*-type arsenite oxidase gene (1). Arx is a recently identified group of arsenite oxidases, originally characterized in chemoautotrophic arsenite oxidizers from Mono Lake (Mono County, CA, USA) (2–5). The haloalkaliphilic strain Alkalilimnicola ehrlichii MLHE-1 catalyzes arsenite oxidation coupled to nitrate reduction (2–4), and the anoxygenic phototroph *Ectothiorhodospira* sp. strain PHS-1 oxidizes arsenite during photosynthesis (6, 7). Therefore, the genome sequence of strain ANAO-440 would provide useful information regarding ArxA-type arsenite oxidation in heterotrophic bacteria.

Strain ANAO-440 was grown as previously described (1), and genomic DNA was extracted using a MoBio power soil kit (Qiagen). A paired-end library (insert size, ∼350 bp) was prepared using a NEBNext Ultra DNA library prep kit for Illumina (New England Biolabs), and genome sequencing was performed on a HiSeq X sequencing platform (Illumina, San Diego, CA) at the National Institute for Environmental Studies. Overall, 14,972,975 raw paired-end reads were generated (150-bp paired-end format), and low-quality sequences (Q ≤ 13) were removed using the Trim_Reads tool implemented in CLC Genomic Workbench (GW) 20.0.2 (Qiagen). The sequences were assembled *de novo* in slow mode in GW using default parameters, except for the minimum contig length (500 bp) and word size (30); the assembly resulted in 64 contigs with an *N*_50_ value of 193,648 bp and a maximum contig length of 449,532 bp. The draft genome sequence of strain ANAO-440 was 4,309,801 bp long with 534.0× genome coverage and a G+C content of 62.6%. Annotation was conducted using the NCBI Prokaryotic Genome Annotation Pipeline (8) and GhostKOALA in KEGG (9), resulting in 3,866 predicted protein-coding sequences, 60 tRNAs, and 5 rRNAs (1 copy of 5S, and 2 copies each of 16S and 23S). BLASTn analysis of the 16S rRNA gene showed that this strain is closely related to other *Halomonas* strains (sequence identity, >97%), such as *Halomonas chromatireducens* strain AGD 8-3 (GenBank accession number CP014226.1).

The draft genome sequence of ANAO-440 contained the *arx* gene island, *arxB2ABCD*, which was previously identified in *Ectothiorhodospira* sp. strain PHS-1 and *A. ehrlichii* MLHE-1 (2, 7). However, the *arxXSR* genes encoding putative regulatory proteins that were found adjacent to the *arxB2ABCD* gene islands in PHS-1 and MLHE-1 (7) were absent from the *arx* island in ANAO-440, possibly indicating the presence of a distinct regulatory mechanism for *arx* genes in this heterotrophic arsenite-oxidizing *Halomonas* strain. Additionally, the functional genes encoding enzymes in the denitrification pathway (*narGHI*, *napAB*, *nirS*, *norBC*, and *nosZ*) were present in the draft genome sequence, while lacking either the dissimilatory arsenate reductase gene *arr* or the *aioA*-type arsenite oxidase gene. The draft genome sequence of *Halomonas* sp. strain ANAO-440 provides valuable information regarding Arx-dependent arsenite oxidation in phylogenetically and physiologically diverse microorganisms.

### Data availability.

The draft genome sequence was deposited in GenBank under accession number JAHXBV000000000, BioProject accession number PRJNA670823, BioSample accession number SAMN20309323, and SRA accession number SRR15204658.
